# Budd-Chiari syndrome from pleomorphic sarcoma compressing the inferior vena cava: A case report

**DOI:** 10.1016/j.radcr.2025.06.081

**Published:** 2025-07-26

**Authors:** Brandon Camp, Clifford N. Danza, Cameron Fateri, Shawn Sun, Michael Phillipi, Roozbeh Houshyar

**Affiliations:** Department of Radiological Sciences, University of California, Irvine, CA 92868-3201, USA

**Keywords:** Budd-Chiari syndrome, Inferior vena cava, Pleomorphic sarcoma, Case report

## Abstract

Budd-Chiari syndrome is a rare condition characterized by obstruction of the hepatic veins or inferior vena cava, leading to portal hypertension and liver dysfunction. While common etiologies include thrombosis or malignancy, we present, to our knowledge, the first reported case in the English-language literature of Budd-Chiari syndrome secondary to metastatic undifferentiated pleomorphic sarcoma. A 52-year-old male with an extensive history of metastatic undifferentiated pleomorphic sarcoma presented with a 10-day history of vomiting and abdominal pain. A contrast-enhanced computed tomography scan of the abdomen revealed a large mediastinal mass representing likely further metastasis compressing the suprahepatic inferior vena cava, resulting in severe congestive hepatopathy consistent with Budd-Chiari syndrome. While hepatocellular carcinoma, renal cell carcinoma, leiomyosarcoma, and other malignancies have been linked to Budd-Chiari syndrome, this report highlights a novel cause. In cases of Budd-Chiari syndrome secondary to malignancy, vessel obstruction may result from compression, infiltration, or bland thrombus formation.

## Introduction

Budd-Chiari syndrome (BCS) is a rare disorder characterized by hepatic venous outflow obstruction, resulting from partial or complete occlusion of the hepatic veins or the intrahepatic segment of the inferior vena cava (IVC) extending to the right atrium. The annual incidence of BCS in the United States is approximately 10 per million and predominantly affects middle-aged women [1]. Clinically, BCS manifests with nonspecific symptoms such as abdominal pain, ascites, hepatomegaly, and jaundice. The most common cause is venous thrombosis, though other etiologies have been identified, including abdominal trauma, surgical procedures, infections, sarcoidosis, vasculitis, and malignancy [1]. Notably, there are significant geographical variations in BCS etiology and presentation. In Western countries, hepatic vein thrombosis, often associated with myeloproliferative neoplasms, is the predominant cause [1,2]. In contrast, Asian populations more commonly exhibit chronic forms of BCS, with membranous obstruction of the IVC or combined IVC and hepatic vein involvement, and a higher proportion of idiopathic cases [2].

Undifferentiated pleomorphic sarcoma (UPS) is a rare, aggressive subtype of soft tissue sarcoma (STS) with a heterogeneous histological appearance. The pathogenesis of UPS remains unclear and may involve multiple complex genetic alterations. UPS is diagnosed by exclusion following histopathological examination and accounts for roughly 10% of adult STS cases [3]. It typically presents between ages 50 and 70, often as a rapidly enlarging, painless mass, with most cases arising in the extremities and trunk (68%) or the abdominal cavity and retroperitoneum (16%) [4]. Despite advancements in the management of STS, the prognosis for UPS remains poor due to its aggressive nature and high metastatic potential [3]. To our knowledge, this is the first reported case in the English-language literature of BCS secondary to compression of the IVC by metastatic UPS.

## Case report

**Fig. 1:**
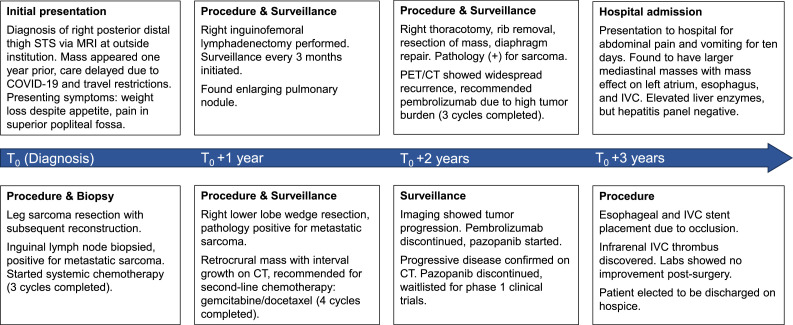
Timeline of patient care. Timeline for patient care from initial presentation to discharge on hospice.

A 52-year-old Asian male with a history of metastatic UPS, status-post resection of tumors in the right lower extremity, lung, and diaphragm, presented with a 10-day history of nausea, vomiting, and abdominal pain. He reported right upper quadrant pain, decreased appetite, a 20-pound unintentional weight loss over the past month, persistent coughing and retching, and bilateral lower extremity edema. His social history was notable for tobacco use. A patient care timeline is shown in Figure 1.

On physical examination, the patient exhibited tenderness in the right-upper abdominal quadrant, with normoactive bowel sounds. Cardiac and pulmonary examinations were unremarkable. His body mass index was calculated at 16.6 kg/m^2^. Initial vital signs showed a blood pressure of 104/77 mmHg, heart rate of 110 bpm, and a temperature of 97.7°F.

**Fig. 2:**
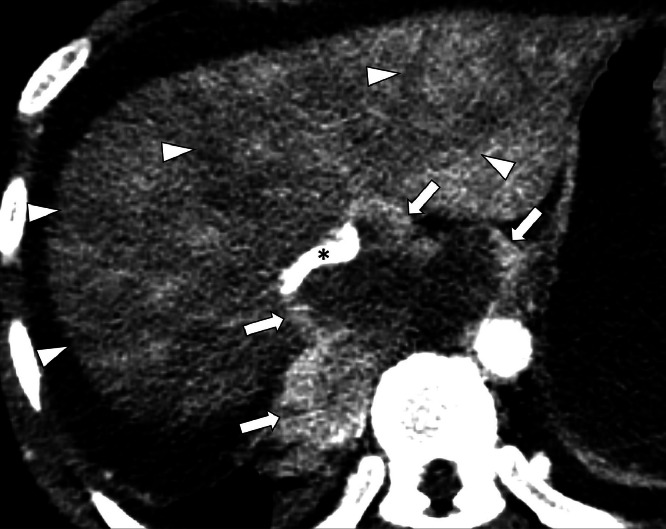
CT of mediastinal mass progression and hepatic venous congestion. Axial contrast enhanced CT of the abdomen shows a large mass in the lower chest (arrows) severely compressing and obstructing the IVC (*). The liver demonstrates heterogeneous enhancement with linear and wedge-shaped areas of decreased enhancement (arrowheads), consistent with hepatic venous congestion.

**Fig. 3:**
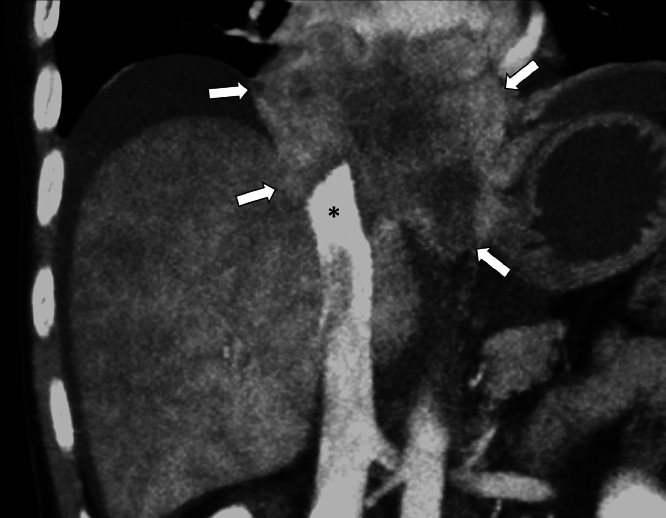
CT of mediastinal mass progression and obstruction. Coronal contrast enhanced CT, maximum intensity projection of the abdomen demonstrates a large mass (arrows) in the lower chest with acute obstruction of the IVC (*).

Laboratory tests revealed elevated liver enzymes, including AST 223 U/L, ALT 270 U/L, ALP 435 U/L, and a total bilirubin of 5.6 mg/dL. A chest X-ray identified a pleural mass in the right upper lobe and a separate mediastinal mass. A contrast-enhanced computed tomography (CT) scan of the chest, abdomen, and pelvis demonstrated an 8.4 cm lobulated mediastinal mass exerting severe compression on the suprahepatic IVC, with findings consistent with hepatic congestion (Fig. 2, Fig. 3). Mass effect exerted on the esophagus and right atrium was also present. Given the extensive history of metastatic UPS in the patient, the mass was presumed to be further metastasis; no additional histopathologic workup was performed.

**Fig. 4:**
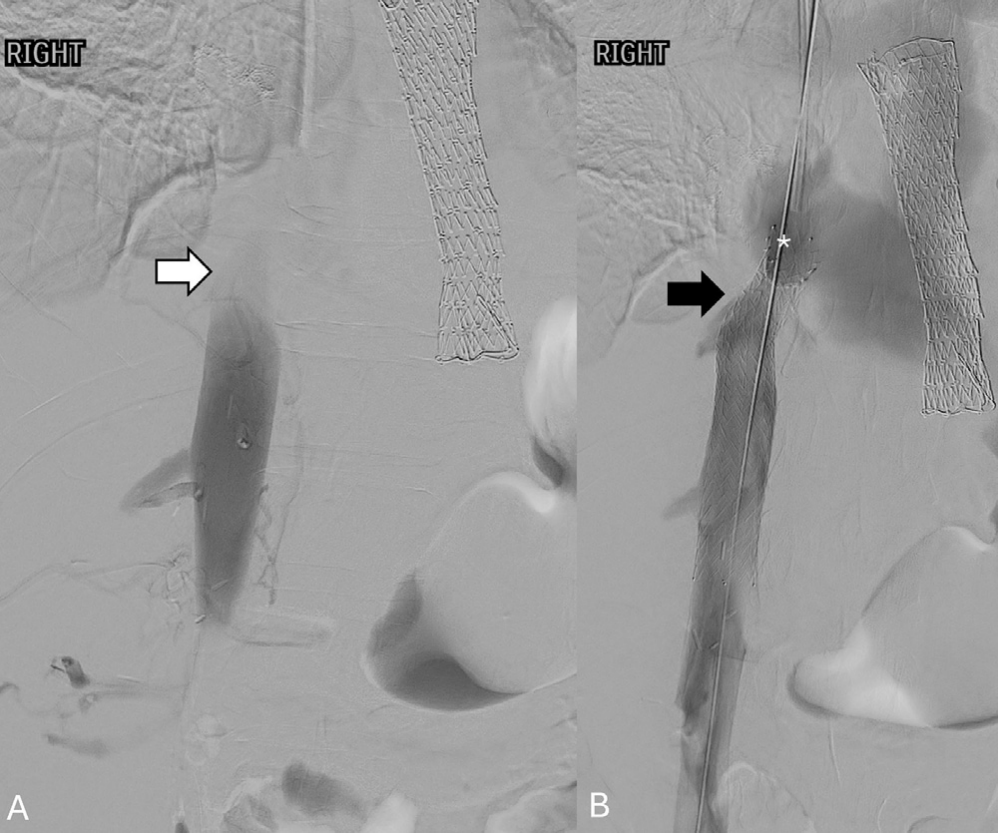
Inferior vena cava venogram demonstrating stent and thrombus. Inferior vena cava venogram demonstrates A) filling defect in the intrahepatic portion (white arrow), consistent with thrombus. Post stenting B) (black arrow), flow is seen extending through the IVC into the right atrium (*).

Given the patient’s poor surgical candidacy, a multidisciplinary team recommended palliative endovascular interventions. On hospital day 2, esophagogastroduodenoscopy was performed with placement of an Endomaxx 19 Fr x 10 cm fully covered metal stent to relieve compression on the esophagus. The following day, interventional radiology placed an IVC stent across the compressed suprahepatic segment under fluoroscopic guidance. During the procedure, an incidental infrarenal IVC thrombus was identified and treated with catheter-directed thrombolysis (Fig. 4). Post-procedurally, the patient was initiated on therapeutic anticoagulation, transitioned from heparin to fondaparinux due to concern for heparin-induced thrombocytopenia following a significant platelet drop and intermediate 4T score.

The patient’s hospitalization was complicated by acute hypoxic respiratory failure, attributed to aspiration during esophageal stent placement and compounded by volume overload. He developed new bilateral pulmonary opacities and leukocytosis, prompting broad-spectrum antibiotics and diuresis. Additionally, he experienced new-onset atrial flutter with rapid ventricular response, successfully managed with adenosine and amiodarone infusion.

Throughout the admission, the patient demonstrated progressive anasarca, scrotal edema, and worsening hepatic dysfunction despite intervention. Nutritional support was provided with oral supplements and total parenteral nutrition, though ultimately de-escalated in the setting of transitioning goals of care. Given his overall disease burden, poor functional status, and lack of durable benefit from further interventions, the decision was made to prioritize comfort-focused care. He was discharged to hospice on hospital day 11.

## Discussion

BCS secondary to metastatic UPS is a novel and previously unreported condition. This case highlights both an uncommon mechanism and unusual anatomical location for malignancy-induced BCS. The patient's symptoms were driven by mass effect on the distal esophagus and significant compression of the IVC, leading to severe congestive hepatopathy. To address these complications, stents were placed in both the esophagus and IVC to relieve the occlusions.

The malignancies most commonly associated with BCS include hepatocellular carcinoma, adrenal carcinoma, renal cell carcinoma, leiomyosarcoma, right atrial myxoma, and Wilms tumor, collectively accounting for approximately 10% of cases; these cancers can induce BCS via direct vessel compression, vascular invasion, or by promoting a hypercoagulable state [5]. Advanced imaging plays a crucial role in identifying the cause of BCS, as it can differentiate bland thrombi, which do not contain cancer cells, from tumor thrombi. Additionally, imaging provides insights into the primary site of tumor origin, which is critical for diagnosis and treatment planning [6]. A systematic evaluation of the IVC is particularly important in cases with suspected neoplastic involvement due to its diagnostic and therapeutic implications.

The primary goal of BCS treatment is to relieve the underlying cause of venous obstruction. Depending on the etiology, acute treatments may include anticoagulation, thrombolysis, stenting, or surgical decompression. In cases associated with hypercoagulability, such as myeloproliferative disorders, long-term anticoagulation is often necessary. Liver transplantation is considered in cases where conservative management fails, or where irreversible liver damage has occurred [5]. Invasive procedures, such as trans jugular intrahepatic portosystemic shunt (TIPS), have been shown to improve outcomes when employed as part of a stepwise treatment approach. While the efficacy of stent placement could not be fully evaluated in this case due to the patient’s rapid clinical deterioration, stenting remains a viable option for certain patients with compressive BCS [7].

Although this report documents a unique presentation of BCS caused by metastatic UPS, the rarity of this malignancy, and of this complication, inherently limits the broader applicability of our findings. Nonetheless, this case serves as a valuable illustration of the need to consider atypical metastatic patterns and mechanical causes of vascular obstruction in patients with advanced STS.

## Conclusion

In conclusion, this report presents the first known case of BCS caused by severe mass effect on the IVC from metastatic UPS. Malignancies associated with BCS may induce vessel compression, infiltration, or thrombus formation, and treatment should prioritize immediate relief of venous obstruction and strategies to prevent recurrence.

## Consent for publication

Written informed consent was obtained from the patient for publication of this case report and accompanying images.

## Author statement

All authors acknowledge that the material presented in this manuscript has not been previously published nor is it simultaneously under consideration by any other journal.

## Patient consent

Written informed consent for the publication of this case report was obtained from the patient.
